# 维奈克拉联合阿扎胞苷治疗NPM1/IDH1突变急性髓系白血病复发患者获持久分子学缓解1例

**DOI:** 10.3760/cma.j.issn.0253-2727.2022.02.017

**Published:** 2022-02

**Authors:** 霄虹 刘, 莹 武, 晓军 黄, 浩 江

**Affiliations:** 北京大学人民医院、北京大学血液病研究所，国家血液系统疾病临床医学研究中心，北京 100044 Peking University People's Hospital, Peking University Institute of Hematology, National Clinical Research Center for Hematologic Disease, Beijing 100044, China

患者，男，66岁。2016年2月因“乏力”就诊。查体：贫血貌，余无明显阳性体征。血常规：WBC 1.12×10^9^/L，HGB 59 g/L，PLT 117×10^9^/L。骨髓象：增生低下，原始细胞30％。流式细胞术免疫分型：异常髓系表型，CD117^+^幼稚细胞占20.47％，表达CD38、CD13、HLA-DR。白血病相关基因检测：WT1转录本水平37.5％，NPM1突变转录本水平2.59％，检测到CEBPA单突变，FLT3-ITD阴性，TP53阴性。染色体核型：46，XY[20]。二代测序：IDH1突变频率26.2％，NPM1突变频率24.6％，SF3B1突变频率42.7％。诊断：急性髓系白血病（AML）伴NPM1突变、IDH1突变、CEBPA单突变、SF3B1突变。

2016年2月12日予CAG（阿糖胞苷10 mg/m^2^ 每12 h 1次，第1～14天；阿克拉霉素20 mg/d，第1～4天；G-CSF 300 µg/d，第1～14天）方案诱导化疗。2016年3月11日血常规恢复，评估骨髓达完全缓解（CR），NPM1突变转录本水平0.0052％。中剂量阿糖胞苷（1 g/m^2^，每12 h 1次，第1～3天）巩固治疗1个疗程后NPM1突变转录本水平0.05％，1个疗程CAG方案后NPM1转阴，随后给予2个疗程MA（米托蒽醌10 mg/d，第1～2天；阿糖胞苷500 mg/d，第1～4天）方案巩固治疗，期间NPM1水平持续阴性，但患者血常规未能完全恢复正常，WBC波动于（2.42～2.95）×10^9^/L，HGB波动于76～100 g/L，PLT波动于（39～53）×10^9^/L。

因每疗程化疗后均出现肺部感染，且巩固治疗期间造血未完全恢复，2016年11月9日停化疗，予干扰素维持治疗1个月，因Ⅳ级血液学不良反应停干扰素治疗，定期监测骨髓，2017年9月15日骨髓NPM1突变转为阳性（0.0004％），2018年1月19日骨髓NPM1水平上升明显（0.11％）。2018年1月30日给予地西他滨联合西达苯胺（地西他滨20 mg/m^2^，第1～5天；西达苯胺20 mg每周两次，共4周）治疗1个疗程后骨髓NPM1水平0.016％。2018年11月5日NPM1水平再次上升（0.26％）。2018年12月4日给予地西他滨序贯NK细胞治疗［地西他滨20 mg/m^2^第1～5天，第8天输注NK细胞（赛傲生物技术有限公司产品，100 ml，细胞数量4.43×10^9^，细胞活率97.8％）］，骨髓NPM1水平0.061％。2019年4月27日骨髓象：增生活跃，原始细胞6.5％。免疫残留：5.27％。NPM1水平6.82％。提示骨髓血液学复发。2019年5月2日血常规：WBC 4.24×10^9^/L，HGB 153 g/L，PLT 87×10^9^/L。

2019年5月3日给予维奈克拉（Ven，德国AbbVie公司产品，自购于中国香港）+阿扎胞苷（AZA）治疗（Ven第1天100 mg，第2天200 mg，第3～25天400 mg；AZA 135 mg，每日1次，第1～7天）1个疗程。治疗期间有轻度恶心，第20天出现Ⅲ级血液学不良反应，第26天停化疗，评估骨髓达血细胞未完全恢复的CR（CRi），停化疗两周造血完全恢复。2019年6月14日（Ven+AZA开始治疗后第42天）骨髓象：增生活跃，原始粒细胞1.5％。免疫残留：阴性。NPM1阴性，提示骨髓获得第2次CR（CR_2_），NPM1水平转阴。2019年6月20日给予患者Ven（Venclyxto，AbbVie Türkiye公司产品）200 mg口服28 d，间隔28 d进行维持治疗，监测骨髓持续CR，NPM1持续阴性。每2～3个月评估骨髓及外周血常规。2021年5月19日骨髓象：增生活跃，未见原始粒细胞。免疫残留：阴性。NPM1阴性。至2021年5月，患者CR_2_后获分子学完全缓解持续2年。

